# Whole-transcriptome analyses of ovine lung microvascular endothelial cells infected with bluetongue virus

**DOI:** 10.1186/s13567-024-01372-0

**Published:** 2024-09-27

**Authors:** Shimei Luo, Yunyi Chen, Xianping Ma, Haisheng Miao, Huaijie Jia, Huashan Yi

**Affiliations:** 1 College of Veterinary Medicine, Southwest University, Rongchang, Chongqing, 402460 China; 2 Chongqing Veterinary Science Engineering Research Center, Rongchang, Chongqing, 402460 China; 3 Yunnan Tropical and Subtropical Animal Virus Disease Laboratory, Yunnan Veterinary and Animal Science Institute, Kunming, 650224 China; 4 State Key Laboratory for Animal Disease Control and Prevention, Lanzhou Veterinary Research Institute, Chinese Academy of Agricultural Sciences, Lanzhou, 730046 China; 5 Immunology Research Center, Medical Research Institute, Southwest University, Rongchang, Chongqing, 402460 China

**Keywords:** Bluetongue virus, ovine lung microvascular endothelial cells, ECM, type I interferon, protein‒protein interaction network

## Abstract

Bluetongue virus (BTV) infection induces profound and intricate changes in the transcriptional profile of the host to facilitate its survival and replication. However, there have been no whole-transcriptome studies on ovine lung microvascular endothelial cells (OLMECs) infected with BTV. In this study, we comprehensively analysed the whole-transcriptome sequences of BTV-1 serotype-infected and mock-infected OLMECs and subsequently performed bioinformatics differential analysis. Our analysis revealed 1215 differentially expressed mRNA transcripts, 82 differentially expressed long noncoding RNAs (lncRNAs) transcripts, 63 differentially expressed microRNAs (miRNAs) transcripts, and 42 differentially expressed circular RNAs (circRNAs) transcripts. Annotation from Gene Ontology, enrichment from the Kyoto Encyclopedia of Genes and Genomes, and construction of endogenous competing RNA network analysis revealed that the differentially expressed RNAs primarily participated in viral sensing and signal transduction pathways, antiviral and immune responses, inflammation, and extracellular matrix (ECM)-related pathways. Furthermore, protein‒protein interaction network analysis revealed that BTV may regulate the conformation of ECM receptor proteins and change their biological activity through a series of complex mechanisms. Finally, on the basis of real-time fluorescence quantitative polymerase chain reaction results, the expression trends of the differentially expressed RNA were consistent with the whole-transcriptome sequencing data, such as downregulation of the expression of COL4A1, ITGA8, ITGB5, and TNC and upregulation of the expression of CXCL10, RNASEL, IRF3, IRF7, and IFIHI. This study provides a novel perspective for further investigations of the mechanism of the ECM in the BTV-host interactome and the pathogenesis of lung microvascular endothelial cells.

**Supplementary Information:**

The online version contains supplementary material available at 10.1186/s13567-024-01372-0.

## Introduction

Bluetongue (BT) is a vector-borne infectious disease of ruminants caused by bluetongue virus (BTV) and is transmitted mainly by *Culicoides*. Among the susceptible animals, sheep exhibit the highest susceptibility to BTV infection, with mortality ranging from 2 to 30%, reaching a maximum of 70%, which significantly affects the healthy development of the sheep industry [1, 2]. Increasing global temperatures increase the range and abundance of vectors and their animal reservoirs and further affect viral development, replication, and survival, resulting in potential threats and significant economic losses to global livestock production [3].

BTV belongs to the genus *Orbivirus* in the family *Reoviridae*. BTV virions are icosahedral and organized as triple-layered capsid that incorporates 10 double-stranded RNA segments encoding seven structural proteins (VP1, VP2, VP3, VP4, VP5, VP6, and VP7) and six non-structural proteins (NS1–NS5 and NS3A) [4–6]. To date, 36 serotypes (BTV-1–BTV-36) have been found worldwide, including 27 notifiable BTV serotypes and additional “atypical” putative serotypes [7]. There are 13 BTV serotypes prevalent in China, namely, BTV-1, -2, -3, -4, -5, -7, -9, -12, -15, -16, -21, -24, and putative BTV-29. The BTV-1 strain (Y863), in particular, caused the first recorded outbreak of BT in Shizong County, Yunnan Province, in 1979 [8]. The prevalence of BTV in southern China has been extensively documented, with clinical cases often associated with asymptomatic infections and the main serotypes identified. Although different serotypes of BTV exhibit similar infectivity in susceptible animals, predicting the risk of clinical infection on the basis of genomic or segment gene variations remains challenging. Furthermore, the new strains of BTV that evolved in the isolated Chinese geographical environment have large mutations in their genomes, resulting in greater complexity of the BTV itself, thereby posing a potential threat to secure animal husbandry [9].

Bluetongue disease is a systemic haemorrhagic viral fever, and its clinical symptoms are derived from direct viral endothelial cell (EC) damage and the host cell response. In particular, pulmonary oedema can be fatal for affected animals. Bluetongue disease is a major disease of sheep and is associated with high mortality and morbidity. The clinical manifestations of BTV infection in cattle and sheep are scientifically supported and associated with endothelial cell infection and the expression of various cytokines. Subsequent investigations have revealed variations in the mechanism of apoptosis and infection kinetics between bovine and ovine pulmonary artery and pulmonary microvascular ECs following BTV infection [10, 11]. Comparative analysis of the responses of primary sheep pulmonary microvascular endothelial cells (OLmVECs) infected with BTV and bovine pulmonary microvascular ECs revealed a direct association between inflammatory mediators and microvascular injury caused by BTV [12]. In recent years, numerous studies have demonstrated that long noncoding RNAs (lncRNAs) modulate the host defence against viral infections by exerting regulatory effects on transcription and post-transcriptional processes. Following viral infection, host cells exhibit differential expression of lncRNAs and cytokines to elicit antiviral responses and mediate inflammatory factor-mediated reactions [13]. Several researchers have conducted transcriptome studies by infecting bovine microvascular endothelial cells (BLmVECs) with BTV, demonstrating that BTV infection induces the activation of BLmVECs and the subsequent production of inflammatory mediators, leading to microvascular injury in ruminants and altering the mechanism of BLmVEC apoptosis [12]. Currently, comprehensive transcriptome analysis of susceptible sheep pulmonary microvascular ECs infected with BTV is lacking. Therefore, we must increase our understanding of the pathological mechanisms underlying BTV infection in sheep lung cells.

Transcriptome sequencing technology (RNA-seq) enables the precise quantification of diverse transcriptome phenotypes, thereby playing a pivotal role in identifying disease biomarkers, elucidating tissue- and cell-specific effects, and studying the spatial localization of disease-related mechanisms [14]. lncRNAs and circular RNAs (circRNAs) modulate the host transcriptomic response and actively participate in diverse cellular processes, including antiviral immunity, metabolic pathways, and cell apoptosis. Additionally, circRNAs function as “sponges” for microRNAs (miRNAs) and participate in competitive endogenous RNA (ceRNA) networks with miRNAs and messenger RNAs, thereby regulating RNA transcription and protein production. Viruses can significantly modulate their life cycle and pathogenicity by manipulating or exploiting this intricate network [15]. Although several studies have examined the transcriptome of susceptible animal cells infected with BTV, our understanding of transcriptional alterations in OLMECs during BTV infection remains ambiguous. The objective of this study was to conduct a comprehensive analysis of the transcriptomes of OLMECs infected with BTV using high-throughput sequencing technology. In this study, we aimed to elucidate the molecular mechanisms involved in BTV infection and understand its influence on the transcriptional profile of these cells, thus revealing the pathogenic mechanism involved.

## Materials and methods

### Strains and cells

The cell lines used in this study were ovine lung microvascular endothelial cells (OLMECs), and the viral strain investigated was BTV serotype 1.

### Cell culture and BTV infection

The cells were resuspended and stored in OLMECs at −80 °C in our laboratory and immersed in a water bath at 37 °C. The samples were subsequently centrifuged at 73 × *g* for 5 min and resuspended in a mixture of 5% foetal bovine serum, 1% triple antibody, and 90% MEM. The cell suspension was evenly spread into T25 cell culture bottles and cultured in a CO_2_ incubator set to 5% CO_2_ and 37 °C. Once the cell density reached approximately 1.2 × 10^5^ cells mL^−1^, sheep microvascular ECs were seeded onto both 12-well and 6-well cell culture plates. These plates were divided into infection and control groups. In the infection group, the cells were inoculated with BTV according to a predetermined infection number (infection number = 1). In contrast, cells in the control group received a virus-free medium as an inoculation treatment. Samples from both groups were collected separately after 36 h for three repeated experiments.

### RNA extraction and qualification

Total RNA from each sample was extracted using TRIzol reagent (Invitrogen, USA). The RNA quantity and purity of each sample were determined using a NanoDrop 2000 spectrophotometer (NanoDrop, USA), ensuring an OD260/280 ratio between 1.8 and 2.2. To assess RNA integrity, gel electrophoresis, a DNF-471 capillary electrophoresis system (Agilent, USA) and an Agilent 5300 Bioanalyzer (Agilent, USA) were used for detection. All six samples had RQN values greater than 9.0, indicating excellent quality of the extracted RNA. These high-quality RNAs were stored at −80 °C for subsequent experiments. In this study, we successfully obtained six long-chain RNA-seq samples with a total of 106.29 GB of clean data; all the samples yielded a minimum of 15.62 GB of clean data with a Q30 base percentage of 93.99% or greater. Additionally, we obtained six small RNA-seq samples with a total raw read count of 80.84 million reads; each sample had more than 13.03 million raw reads and a Q30 base percentage of 96.95% or higher.

### Library construction and sequencing

This study used the Illumina^®^ Stranded Total RNA Prep and Ligation with Ribo-Zero Plus kit (Illumina, USA) to construct sequencing libraries for simultaneous mRNA, lncRNA, and circRNA sequencing using a ribosomal RNA-specific method. The experimental procedures followed microbiome protocols. Total RNA was extracted from the cell samples, and the obtained RNA was subjected to concentration, purity, and integrity tests. To obtain a more comprehensive lncRNA profile, a library was constructed by depleting rRNA, as some lncRNAs in cells lack conventional poly A tails. Reverse transcriptase was used to synthesize an A cDNA strand using mRNA as the template and random primers. During two-strand synthesis, dUTP replaced dTTP in the dNTP reagent to ensure that the second strand of cDNA contained all four bases (A/U/C/G). A base was appended at the 3′ end of the double-stranded cDNA to facilitate Y-shaped splice junction formation. Before polymerase chain reaction (PCR) amplification, UNG enzyme digestion selectively removes the second strand of cDNA, resulting in a library comprising only first-strand cDNAs. Subsequently, PCR amplification and purification were performed using this library. Quantification was conducted using Qubit 4.0, followed by data-based mixing for cluster generation through bridge PCR amplification on the cBot platform. Finally, sequencing analysis was performed using the Illumina NovaSeq 6000 platform (Shanghai, China). The obtained sequencing data were subjected to differential gene expression analysis to identify genes whose expression significantly differed between the infected and control groups.

### Bioinformatics analyses

To ensure the accuracy of subsequent bioinformatics analysis, we used fastp software [16] to process the raw sequencing data by removing adapter sequences, filtering out reads that were self-ligated without any insertions, and trimming the low-quality bases (quality value < 20) at the 3' end. If there were still bases with quality values < 10, the sequence was deleted; otherwise, the sequence was retained. Reads with an N ratio > 10% were removed; sequences with adapters and lengths < 20 bp were discarded to obtain high-quality sequencing data (clean data), ensuring reliable subsequent analysis; and the clean data (reads) obtained after quality control were aligned to the reference genome via HISAT2 software [17]. The reference genome source used in this study was Ovis_aries, which can be accessed at Ensembl tools [18]. Mapped data (reads) were obtained for subsequent transcript assembly and expression level calculations [19]. Known lncRNAs were identified by comparing the merged transcripts with reference transcripts, which were annotated as lncRNAs in the GFF/GTF and lncRNA databases, using default parameters for the mapped clean reads. Additionally, novel lncRNA transcripts were identified on the basis of their length (> 200 bp) and exon number (≥ 2). CNCI [20] (score < 0), CPC [21] (score < 0.5), CPAT [22] (score < 0.5), and Pfam (e-value < 1e−3 no pass) were subsequently employed to predict the protein-coding potential of these transcripts. Ultimately, we selected the intersection of both nonprotein-coding potential results as a novel lncRNA. CircRNAs, characterized by closed-loop structures that confer resistance to degradation and insensitivity to RNA exonucleases, exhibit increased stability in expression. It is increasingly recognized as a valuable biomarker that exerts regulatory effects on mRNA expression through sponge mechanisms. The formation of circRNAs primarily involves reverse connections between downstream exonic splicing donor sites and upstream splicing acceptor sites. Consequently, although linear alignment software can be employed to map linear RNA fragments onto the genome, direct alignment of reads at the back-splice junction (BSJ) of circRNAs is not feasible. CIRI2 software-based analysis with BSJ reads enables the potential identification of circRNAs [23]. RSEM software [24] was used to quantify the expression levels of mRNA and lncRNA, which were subsequently normalized to obtain FPKM values. In contrast, circRNA expression was normalized on the basis of BSJ reads to acquire the RPM values. Following the determination of gene read counts, differential gene expression analysis was performed across multiple sample (≥ 2) projects, enabling the identification of differentially expressed genes. The functional characterization of these differentially expressed genes was subsequently conducted using DESeq2 software [25]. The default filtering criteria were determined on the basis of significantly differentially expressed transcripts, with an FDR < 0.05 and | log_2_FC |> 1. When a gene satisfies both conditions, it is classified as a differentially expressed transcript (DET).

### Construction of ceRNA networks

On the basis of these targeting relationships, a lncRNA (circRNA)-miRNA‒mRNA ceRNA network was constructed with the hub prognostic mRNA as the core. The ceRNA hypothesis reveals a new mechanism for RNA interaction and presents a unique mode of gene expression regulation. It does not introduce new RNA molecules but refers to the existence of multiple types of noncoding RNAs in the complex transcriptional regulatory network in organisms. The network mainly comprised mRNA, lncRNAs, pseudogenes, and circRNAs. These RNAs contain miRNA response elements (MREs) that can be bound by miRNAs and play functional roles. These RNAs compete with each other and co-bind to miRNAs by sharing MREs, forming a complex and finely regulated relationship. By assessing the number of shared MREs and the expression correlation between these RNAs, lncRNA‒miRNA-mRNA and circRNA-miRNA‒mRNA regulatory networks can be established.

### Target gene prediction and functional analyses

To further investigate the functionality of differentially expressed RNA (dif-RNA), we conducted an analysis that predicted the regulatory effects of dif-lncRNAs and dif-miRNAs on their potential target mRNAs. lncRNAs exhibit extensive and intricate functional mechanisms, which can be categorized into cis- and trans-acting mechanisms on the basis of their distinct modes of action. Comprehensive analyses, including CPC, CNCI, CPAT, and Pfam protein domain assessments, revealed that the CPC score remained below 0.5. The CNCI score was < 0, and the CPAT score was < 0.5. lncRNA overlap analysis in the Pfam database was conducted without annotation. The intersection of these software results served as the foundation for further analysis of the newly predicted lncRNAs. mRNAs located within a 100 kb region upstream or downstream of dif-lncRNAs were considered potential trans-targets. The prediction of trans-target genes is based on the principle that both lncRNAs and coding genes influence them because they are correlated with their expression. miRNAs regulate the post-transcriptional processes of target genes. Functional investigations of miRNAs involve the study of the functionality of their target genes. Importantly, a single miRNA can regulate multiple target genes, whereas various miRNAs may also regulate a specific target gene. In animals, miRNAs bind tightly to the 3' noncoding region (3' UTR) of the target gene through the seed sequence (5' terminal 2–8 nt), inhibiting the translation of the target mRNA. The miRanda software [26] was used to predict dif-miRNA. Gene Ontology (GO) resources were used to annotate target mRNAs or host mRNAs of differentially expressed mRNA (dif-mRNA) and dif-lncRNA, dif-miRNA, and dif-circRNA [27]. Additionally, Kyoto Encyclopedia of Genes and Genomes (KEGG) enrichment analysis was employed to investigate the advanced functionalities and practicality of biological systems [28]. The GOplot R (v1.0.2) and ggplot2R (v3.3.6) software packages were used to visualize GO bubble plots and KEGG scatter plots, respectively, with statistical significance determined using a *p* value of < 0.05 [29, 30].

### Protein–protein interaction network and module analysis of dif-mRNAs

The interactions among interacting genes and proteins were analysed via the Search Tool for the Retrieval of Interacting Genes/Proteins online database (v11.5) [31]. The species was set as sheep, and an interaction score > 0.4 was chosen as the cut-off value. Cytoscape software (v3.8.2) was used to visualize the protein‒protein interaction (PPI) network [32]. The tightly interconnected modules were extracted from the PPI network via Cytoscape’s Molecular Complex Detection (MCODE) plug-in, with a degree cut-off of 2, a node score cut-off of 0.2, a K score of 2, and a maximum depth of 100. Enrichment analysis was conducted on mRNAs whose expression was downregulated or upregulated within the PPI network.

### Real-time quantitative PCR

Real-time quantitative PCR (qRT-PCR) was performed using an ABI QuantStudio 3 Quantitative RT-PCR instrument (ABI, USA). After control OLMECs were collected and subjected to 36 h of treatment, the medium was aspirated, and the cells were washed three times with 1 × PBS. Total cellular RNA was extracted using TRIzol™ reagent and reverse-transcribed to cDNA as a template. HiScriptIII RT SuperMix (+ gDNA wiper) and ChamQ Universal SYBR qPCR Master from Vazyme Biomedical Technology were used for verification using real-time qRT-PCR. Table 1 lists the primers used in this study. The reaction conditions were as follows: 95 °C for 2 min and 40 cycles at 95 °C for 5 s, 60 °C for 30 s, 95 °C for 5 s, and 60 °C for 5 s. Relative gene expression was normalized to the expression of β-actin as a standard.

**Table 1:** **Real-time fluorescent quantitative PCR primers**

GenBank	Target genes	Primer sequences (5′–3′)	
NM_007393.5	β-Actin	Forward	CCACTGTCGAGTCGCGTCC
		Reverse	ATTCCCACCATCACACCCTGG
HM017954	CXCL10	Forward	GATTCCTGCAAGTCAAT
		Reverse	TTATGCCTCTTTCTGTG
DQ152970	IRF3	Forward	CATTTCCAACAGCCAACC
		Reverse	CCAGGGACACTGAATACCA
XM_004004655	IFIH1	Forward	GGTCAGCACGAGGAATAA
		Reverse	CTGTGGTAGCGATAAGCA
XM_004013850	RNASEL	Forward	CATGGAGCCGTAGAAG
		Reverse	CAGAAGTGTCAGCGATT
XM_027973786	COL4A1	Forward	TGGGATTGGATTTCCTGGGC
		Reverse	CTCCGACGTTTCCCTTCTCC
NM_001166180	MMP2	Forward	AACGCCATCCCTGATAACC
		Reverse	TTCCGAACTTCACGCTCTT
XM_027971492	THBS4	Forward	TCAGCAACCCGAACCAGTC
		Reverse	CCAATCCCATCCTTGTCAGT
XM_027971473	F2R	Forward	TCGTATTCGTGGTTGGTT
		Reverse	ATGAAGAGGATGGAGCAGTA
NM_001009358	CXCL1	Forward	GCAGAGCGTGAAGGTGAC
		Reverse	GGAGCTGGCCTGGTTTAG
AY656797	TGFB2	Forward	CCCCAGAAGACTACCTCG
		Reverse	AGTATTCCTCGTCGCTCC
NM_001009263	IL18	Forward	CTGGCTGTAACCATCTC
		Reverse	GTCCTGGAACACTTCTC
XM_004003220	PTX3	Forward	CTATCGGTCCATAATGCTTG
		Reverse	TCTTTGAATCCCAGGTGC
XM_042242806	TNC	Forward	CTGGACCGCTACTGATGGTG
		Reverse	GAGAGGCTCAGCTACTGTGG
XM_027960110	ITGA8	Forward	AAGTCAGGTCACGGCTTTGG
		Reverse	TGCCTTCTGGGAGTTTAGCTG
XM_027956587	ITGB5	Forward	ATGCCTGCTAATCCACCCAA
		Reverse	TAATCCTCCACCTGCCGAAC
XM_042232196	PARP9	Forward	AGGAGCAAGTCCTTTGGAGC
		Reverse	TGGAAGGCAGCCATGAGAAG
XM_012177568	Bst-2B	Forward	TTGGCGAACTTGAACCAACAAG
		Reverse	CTTGCGGGTGATGGACACAA
NM_001277168	CXCR4	Forward	TGCTGTGGCAAACTGGTACT
		Reverse	CCGGTCCAGACTGATGAAGG
XM_027960293	IFIT3	Forward	GGCGGCTGAATGCTATGAGA
		Reverse	CTTCATGCTCAGTTGCTGGC

### Statistical analysis

The data obtained using qRT-PCR were analysed via the 2^−ΔΔCt^ method, and significant differences were determined via Student’s t test. The data are presented as the mean ± SD, with an error bar representing at least three independent experiments. In addition, Student’s *t* test was performed for statistical analysis using GraphPad Prism 7.0 software (GraphPad Software, USA). Statistical significance is shown as *p* values: *p < 0.05, **p < 0.01, and ***p < 0.001, and ns represents not significant.

## Results

### Cell infection and harvest

**Figure 1:**
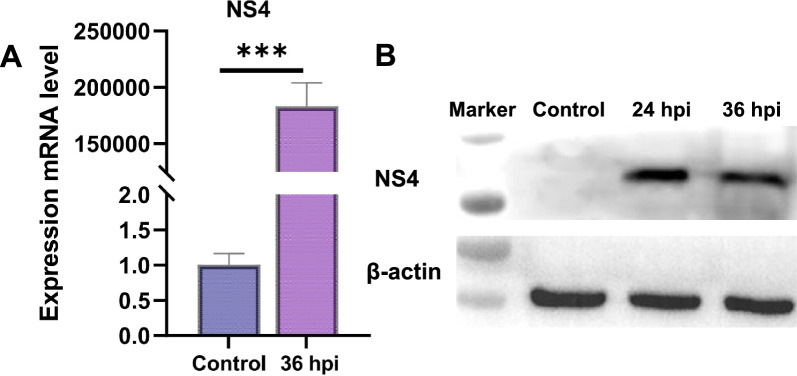
**Validation of BTV-1 infection in OLMECs at 0, 24, and 36 dpi. A** qRT-PCR was performed to verify the successful infection of BTV-1. **B** Western blotting (WB) was used to verify the successful transfection of BTV-1 in OLMECs. The infection status of the BTV-1-infected and control groups at different time points is presented as the mean Ct value ± SD from three independent experiments. NS4 was used as a control for successful infection. In contrast, β-actin served as an internal control.

OLMECs were infected with BTV-1 at a multiplicity of infection of 1, and infection was confirmed by monitoring virus replication using qRT-PCR at 0, 24, and 36 h post-infection (hpi). As depicted in Figure 1A, the expression level of NS4 was significantly upregulated at 36 hpi (*p* < 0.001). The results of viral replication were visualized through the detection of viral NS4 protein bands by western blot (WB) analysis (Figure 1B). OLMECs harvested at 36 hpi subsequently underwent whole-transcriptome sequencing.

### Analysis of differential expression profiles

The expression of the transcript was significantly upregulated, with a fold change (FC) ≥ 2 and a *p* value < 0.05, whereas it was significantly downregulated, with an FC ≤ 0.5 and a *p* value < 0.05. Differential transcripts between the BTV-infected and control groups were identified and subjected to statistical analysis to construct a volcano plot, which visually represented the differential transcripts between these two groups.

**Figure 2:**
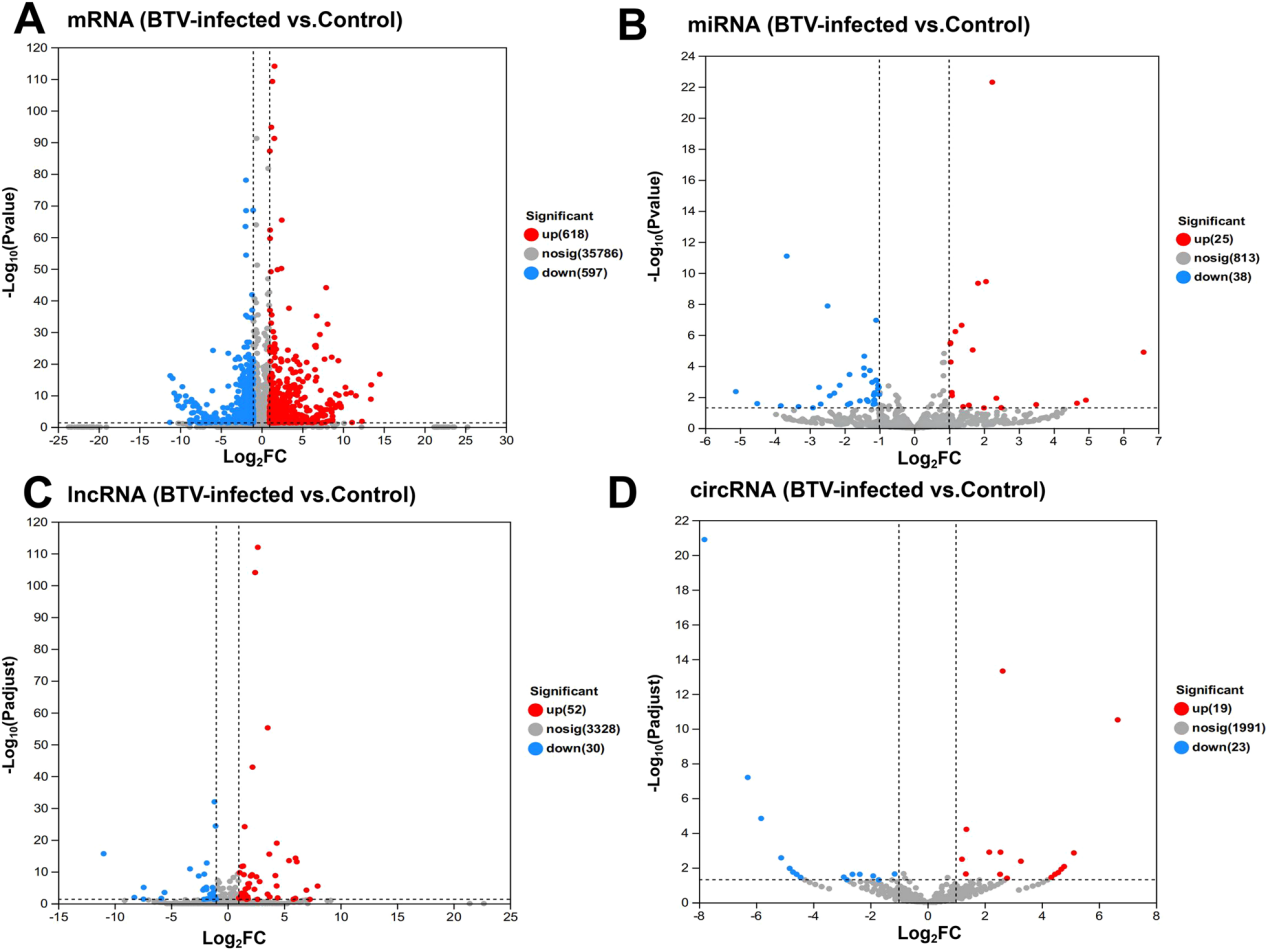
**Volcano plot showing the statistical significance versus magnitude of the log**_**2**_**-fold change. A** Volcano plot of dif-mRNA levels between BTV-1-infected OLMECs and controls. **B** Volcano plot of dif-miRNAs between BTV-1-infected OLMECs and controls. **C** Volcano plot of dif-lncRNAs between BTV-1-infected OLMECs and control OLMECs. **D** Volcano plot of dif-circRNAs between BTV-1-infected OLMECs and controls. Each dot represents a transcript; red dots represent upregulated dif-RNAs, and blue dots represent downregulated dif-RNAs. The total number of upregulated and downregulated dif-RNAs is marked on the right side of each figure.

Compared with the control group, in the BTV-infected group, there were 1215 DET genes at the mRNA level, with 618 upregulated and 597 downregulated transcripts (Figure 2A). Among the differentially expressed lncRNAs, 82 were differentially expressed, of which only eight are known lncRNAs (Figure 2B and Additional file 1). Specifically, the expression levels of 52 lncRNAs were upregulated, whereas those of 32 lncRNAs were downregulated. In our analysis, we identified 63 differentially expressed miRNAs, of which 25 were upregulated and 28 were downregulated (Figure 2C). We detected a set of distinctively expressed circRNAs with a total count of 42; specifically, 19 circRNAs were upregulated, whereas another set comprising 23 circRNAs was downregulated (Figure 2D).

**Figure 3:**
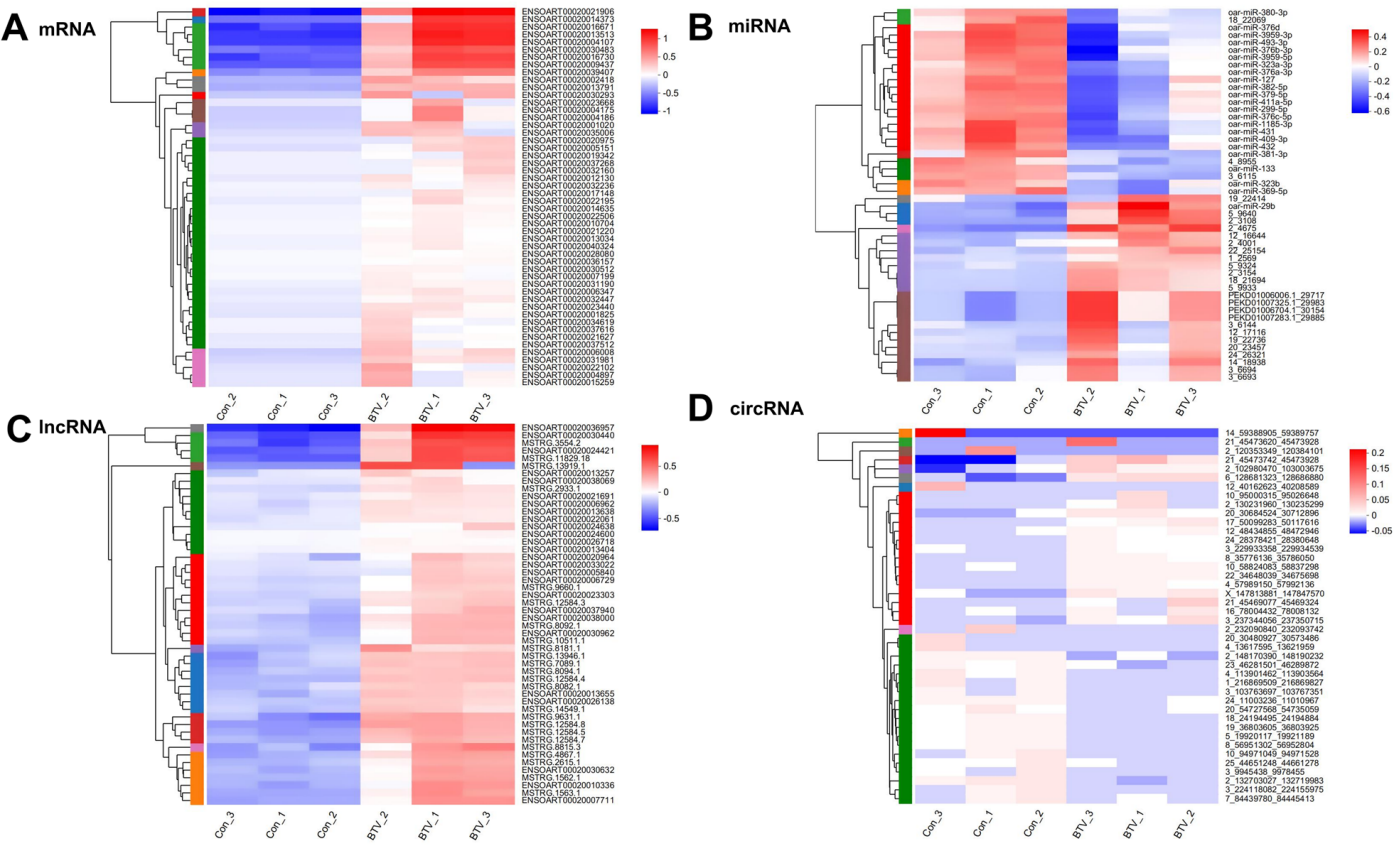
**Hierarchical cluster analyses of dif-RNAs between BTV-1-infected OLMECs and controls. A** Heatmap of the 50 most variable dif-mRNAs. **B** Heatmap of the 50 most variable dif-miRNAs. **C** Heatmap of the 50 most variable dif-lncRNAs. **D** Heatmap of the 42 most variable dif-circRNAs. The colour blocks from blue to red represent dif-RNA expression levels from downregulated to upregulated. The dif-RNAs are labelled to the right of the corresponding heatmaps.

To investigate the expression of DETs between the BTV-infected and control groups and explore potential correlations, we identified the top 50 differentially expressed mRNAs, miRNAs, lncRNAs (dif-mRNAs, dif-miRNAs, and dif-lncRNAs), and 42 dif-circRNAs on the basis of inconsistent expression levels across samples. Subsequently, cluster analysis was performed to generate a heatmap depicting differential RNA expression patterns. Notably, a significant difference was observed between the infected and control groups (Figures 3A–D).

Compared with the control group, the BTV-infected group presented elevated expression levels of several dif-RNAs involved in host antiviral activity, inflammatory responses, and immune responses. These include ENSOART00020030483 (IFII44), ENSOART00020021906 (ISG15), ENSOART00020014373 (PARP9), ENSOART00020016671 (BST-2B), ENSOART00020013513 (IFIT3), ENSOART00020004107 (MX2), ENSOART00020016730 (BST-2A), and ENSOART00020009437 (EPSTI1) (Figure 3A and Additional File 2).

The expression of dif-miRNA was significantly different between the BTV-infected and control groups in this study. Notably, oar-miR-29b, 5_9640, and PEKD01006704.1_30154 were predominantly expressed in the infected group, whereas oar-miR-493-3p, oar-miR-376d, and oar-miR-431 were primarily expressed in the control group.

### Functional enrichment analysis of dif-RNAs (GO)

To better elucidate the functionality of potential differential transcripts between BTV-infected and control samples, as well as to enhance comprehension of the possible role of OLMECs in generating antiviral factors during BTV infection, we performed GO enrichment analysis encompassing three major ontologies: biological process (BP), cellular component (CC), and molecular function (MF).

**Figure 4:**
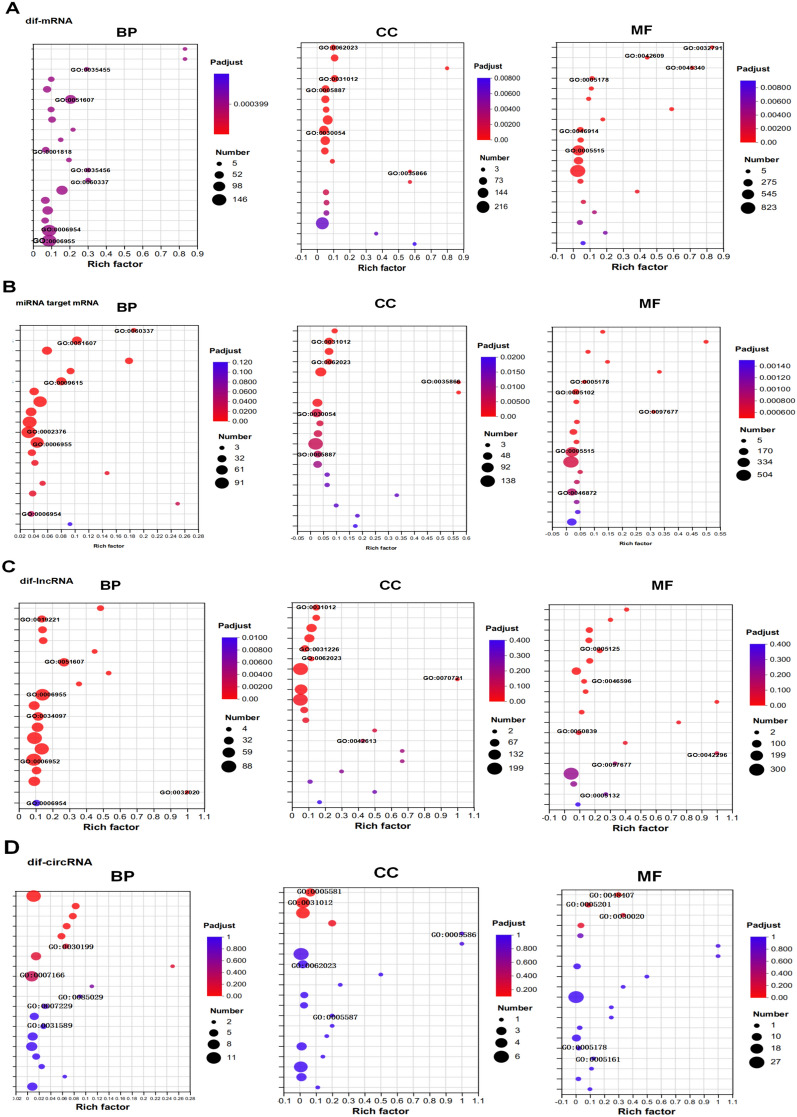
**GO annotations of dif-RNAs under the BP, CC, and MF ontologies. A** GO annotations of dif-mRNAs under the BP, CC, and MF ontologies. **B** GO annotations of the target mRNAs of dif-miNRAs under the BP, CC, and MF ontologies. **C** GO annotations of the target mRNAs of dif-lncNRAs under BP, CC, and MF ontologies. **D** GO annotations of the host mRNAs of circRNAs in the BP, CC, and MF ontologies. Rich factor: The enrichment factor is calculated by dividing the number of genes belonging to the term in the target gene set by the number of all genes in the term in the background gene set. The greater the Rich factor is, the greater the degree of enrichment. The dot size reflects the number of genes/transcripts associated with a given GO term, while the dot colour corresponds to different p.adjust ranges.

In this study, we performed GO enrichment analysis of DETs between the BTV-infected and control groups. The BP ontology contained 1641 enriched terms, whereas the CC and MF ontologies contained 105 and 204 enriched terms, respectively. On the basis of the p. adjust ranking in ascending order, we selected the top 20 enrichment results for visualization. Notably, within the BP and MF ontologies, dif-RNAs were predominantly associated with response to IFN-alpha (GO:0035455), defence response to virus (GO:0051607), type I IFN signalling pathway (GO:0060337), inflammatory response (GO:0006954), protein binding (GO:0005515), and ion binding (GO:0043167), as shown in Figure 4A and Additional file 3.

As miRNAs primarily exert their function through post-transcriptional regulation of target genes, investigating the role of miRNAs involves studying the functions of their target genes. By employing predictive algorithms to identify the mRNA targets of 63 differentially expressed miRNAs (dif-miRNAs), we identified 1905 potential target genes. GO enrichment analysis revealed significant enrichment in key pathways and functions such as the type I IFN signalling pathway (GO:0060337), defence response to the virus (GO:0051607), basement membrane (GO:0005604), and STAT family protein binding (GO:0097677) under the three major ontologies of BP, CC, and MF (Figure 4B and Additional file 4).

lncRNAs play pivotal and intricate roles in diverse biological processes. On the basis of their distinct mechanisms of action, lncRNAs can be categorized into cis- and trans-acting types. By employing dif-lncRNA target gene prediction, we identified 20 dif-lncRNAs that influence the expression of 129 neighbouring genes through cis-regulation. In terms of BP ontology, these target genes primarily participate in the regulation of viral genome replication (GO:0045069), the defence response against viral infection (GO:0051607), the response to the virus (GO:0009615), and the immune response (GO:0006955). With respect to the CC and MF ontologies, these target genes are predominantly involved in extracellular matrix (ECM) organization (GO:0031012), cytokine activity (GO:0005125), and chemokine activity (GO:0008009) (Figure 4C and Additional file 5). The linear homologous genes of 42 differentially expressed circRNAs were subjected to GO enrichment analysis. The linear cognates exhibited significant associations with collagen fibril organization (GO:0030199), extracellular matrix assembly (GO:0085029), the extracellular matrix (GO:0031012), the collagen type IV trimer (GO:0005587), platelet-derived growth factor binding (GO:0048407), and extracellular matrix structural constituents (GO:0005201) in the BP, CC, and MF ontologies (Figure 4D and Additional file 6).

### Pathway analysis of DET based on dif-RNAs (KEGG)

KEGG is a comprehensive database used to classify and quantify signalling pathways and functions in which transcripts are most likely to participate. By analysing these signalling pathways, we can better understand their genetic roles in BF. To further investigate the interaction between viruses and hosts as well as to determine relevant biochemical metabolism and signal transduction processes, we conducted Kyoto Encyclopedia of Genes and Genomes (KEGG) pathway enrichment analysis on dif-mRNAs, dif-miRNA-targeted mRNAs, and dif-lncRNA-targeted mRNAs.

**Figure 5:**
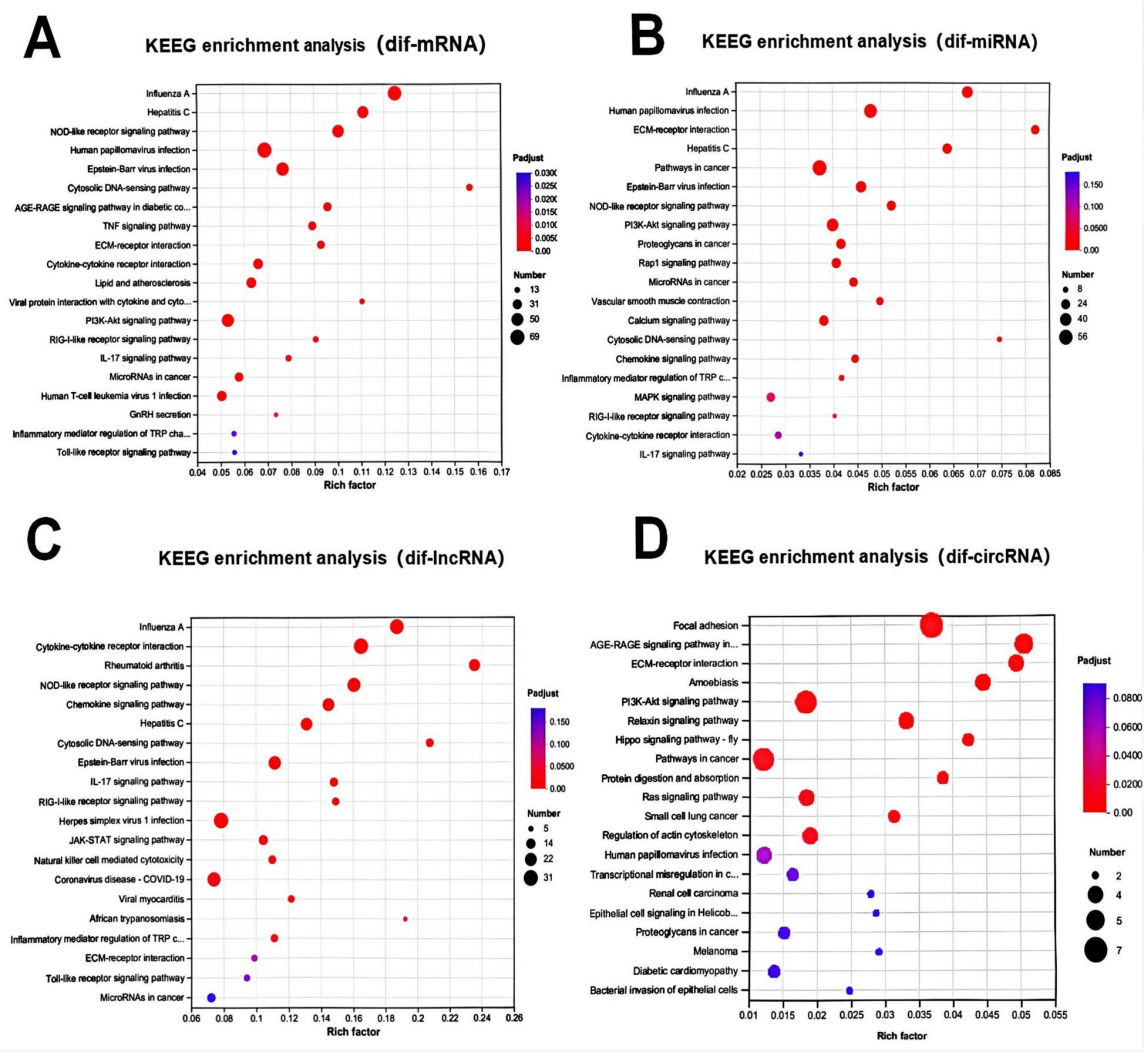
**Top 20 KEGG pathways. A** KEGG enrichment of dif-mRNAs. **B** KEGG enrichment of the target mRNAs of dif-miRNAs. **C** KEGG enrichment of the target mRNAs of dif-lncRNAs. **D** KEGG enrichment of the host mRNAs of circRNAs. The rich factor indicates the ratio of dif-mRNAs enriched in a specific pathway. The size and colour of the solid circles represent the number of enriched dif-mRNAs and the significance of enrichment, respectively.

The dif-mRNAs were significantly enriched in the influenza A, NOD-like receptor signalling, and RIG-I-like receptor signalling pathways. A similar pattern was observed for the TNF signalling pathway (Figure 5A).

The target genes of dif-miRNAs were enriched primarily in ECM-receptor interactions, the cytosolic DNA-sensing pathway, and the regulation of lipolysis in adipocytes (Figure 5B).

The dif-lncRNA-targeted genes were predominantly involved in viral protein interactions with cytokine and cytokine receptors, rheumatoid arthritis, the cytosolic DNA-sensing pathway, influenza A, and the TNF signalling pathway (Figure 5C). KEGG enrichment analysis of 42 differentially expressed linear circRNA transcripts revealed that they were enriched mainly in focal adhesion, ECM–receptor interaction, and the PI3K-Akt signalling pathway (Figure 5D).

### ceRNA network construction and enrichment analyses

**Figure 6:**
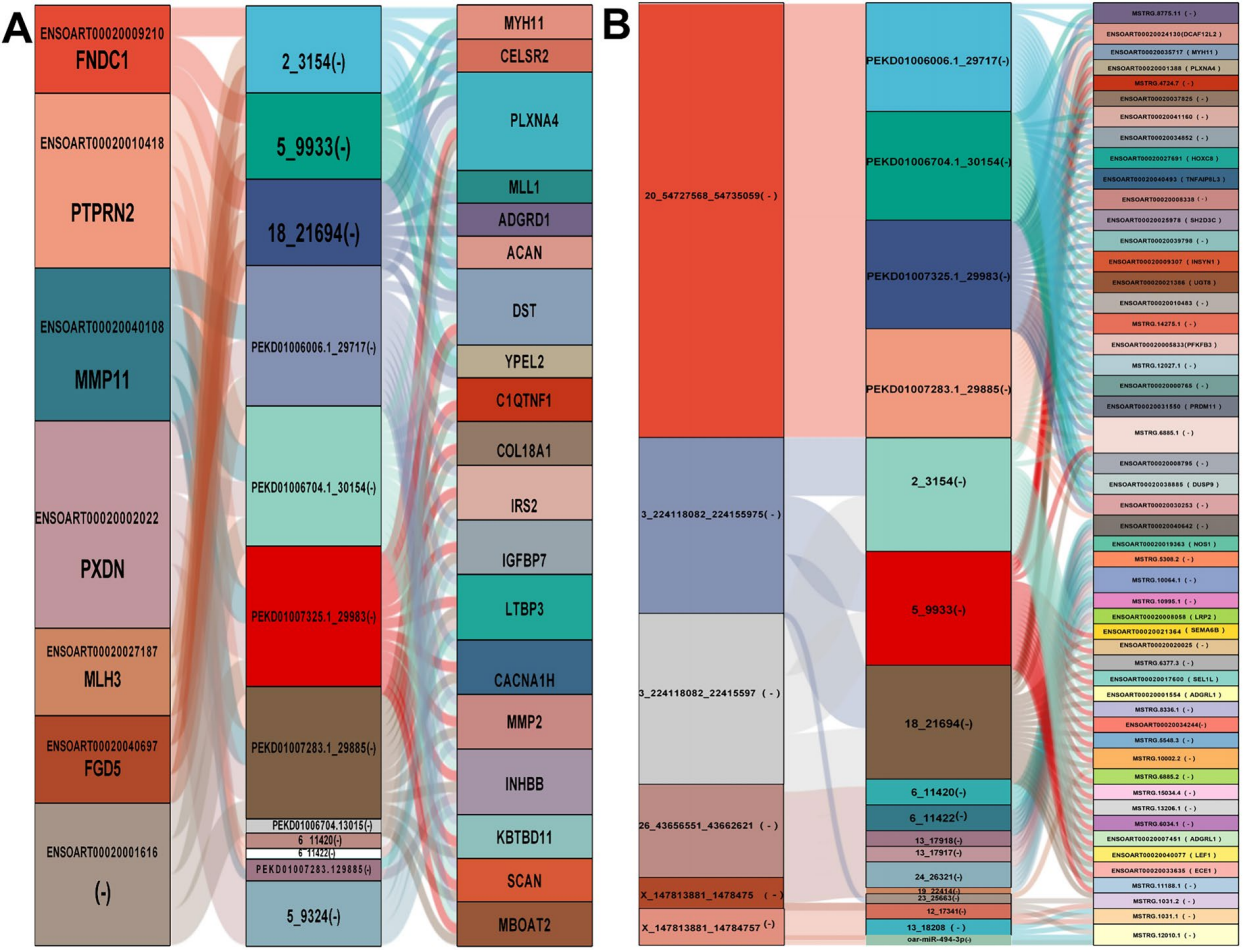
**Sankey diagram of the ceRNA network based on A** lncRNA‒miRNA-mRNA and **B** circRNA-miRNA‒mRNA interaction pairs. Different colour blocks represent different dif-RNA nodes. The flow chart from dif-lncRNAs or dif-circRNAs to dif-miRNAs and finally to dif-lncRNAs or circRNAs regulates mRNA expression through different miRNAs (“–” represents NCBI failure to the corresponding gene name).

**Figure 7:**
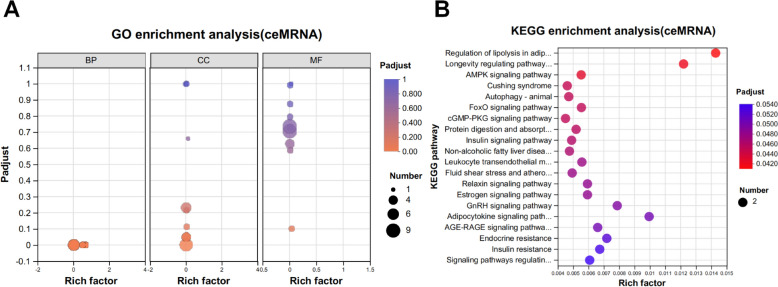
**GO annotation and KEGG enrichment of dif-mRNAs participating in the ceRNA network. A** The size of the solid circle represents the amount of dif-mRNAs enriched under a specific GO condition. GO:1,901,652: response to peptide, GO:0005615: extracellular space, GO:0030312: external encapsulating structure, GO:0005576: extracellular region, GO:0043169: cation binding, GO:0046872: metal ion binding, and GO:0005509: calcium ion binding. **B** The top 20 KEGG pathways were selected for display. The rich factor indicates the ratio of dif-mRNAs enriched in a specific pathway. The size and colour of the solid circles represent the number of enriched dif-mRNAs and the significance of enrichment, respectively.

According to the ceRNA theory, we constructed lncRNA‒miRNA-mRNA and circRNA-miRNA‒mRNA ceRNA networks, in which lncRNAs or circRNAs were used as bait, miRNAs served as core regulators, and mRNAs acted as targets. On the basis of our screening criteria, seven differentially expressed lncRNAs and three differentially expressed circRNAs were selected for this study. Furthermore, we identified 23 differentially expressed mRNAs that were regulated primarily by 12 differentially expressed miRNAs (Figures 6A and B). The heatmap represents the expression patterns of several dysregulated RNAs between the infected and control groups (Figure 3). The regulated genes in the ceRNA network were enriched predominantly in CC and MF ontologies, including extracellular region (GO:0005576), outer packaging structure (GO:0030312), cation binding (GO:0043169), and metal binding (GO:0046872). The KEGG pathway analysis revealed that the target genes were predominantly enriched in several signalling pathways, including the cGMP-PKG signalling pathway, the GnRH signalling pathway, the AGE-RAGE signalling pathway in diabetic complications, the AMPK signalling pathway, the TGF-β signalling pathway, the stem cell pluripotency regulation pathway, miRNAs in the cancer pathway, the lysine degradation pathway, leukocyte migration through the EC pathway, cytogenetic–cytokine receptor interactions, and glycerophospholipid metabolism (Figure 7B and Additional file 7).

### Protein–protein interaction network, module extraction, and functional enrichment analyses

**Figure 8:**
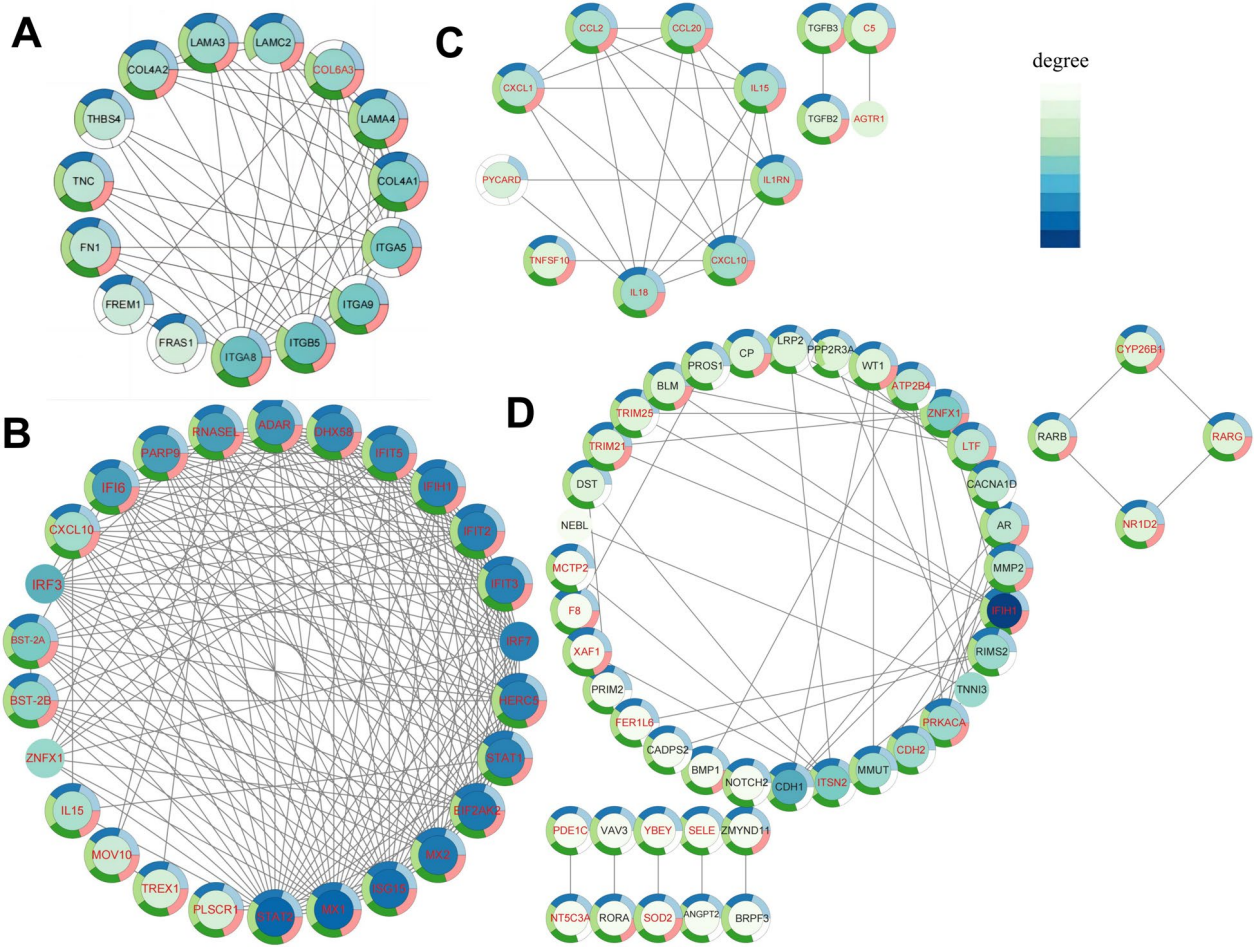
**Modules (A**–**D) extracted from the PPI network.** The MCODE plug-in in Cytoscape was applied to extract densely connected modules from the PPI network with a degree cut-off of 2, a node score cut-off of 0.2, a K score of 2, and a maximum depth of 100. Red and black font represent upregulated and downregulated dif-mRNAs, respectively.

**Figure 9:**
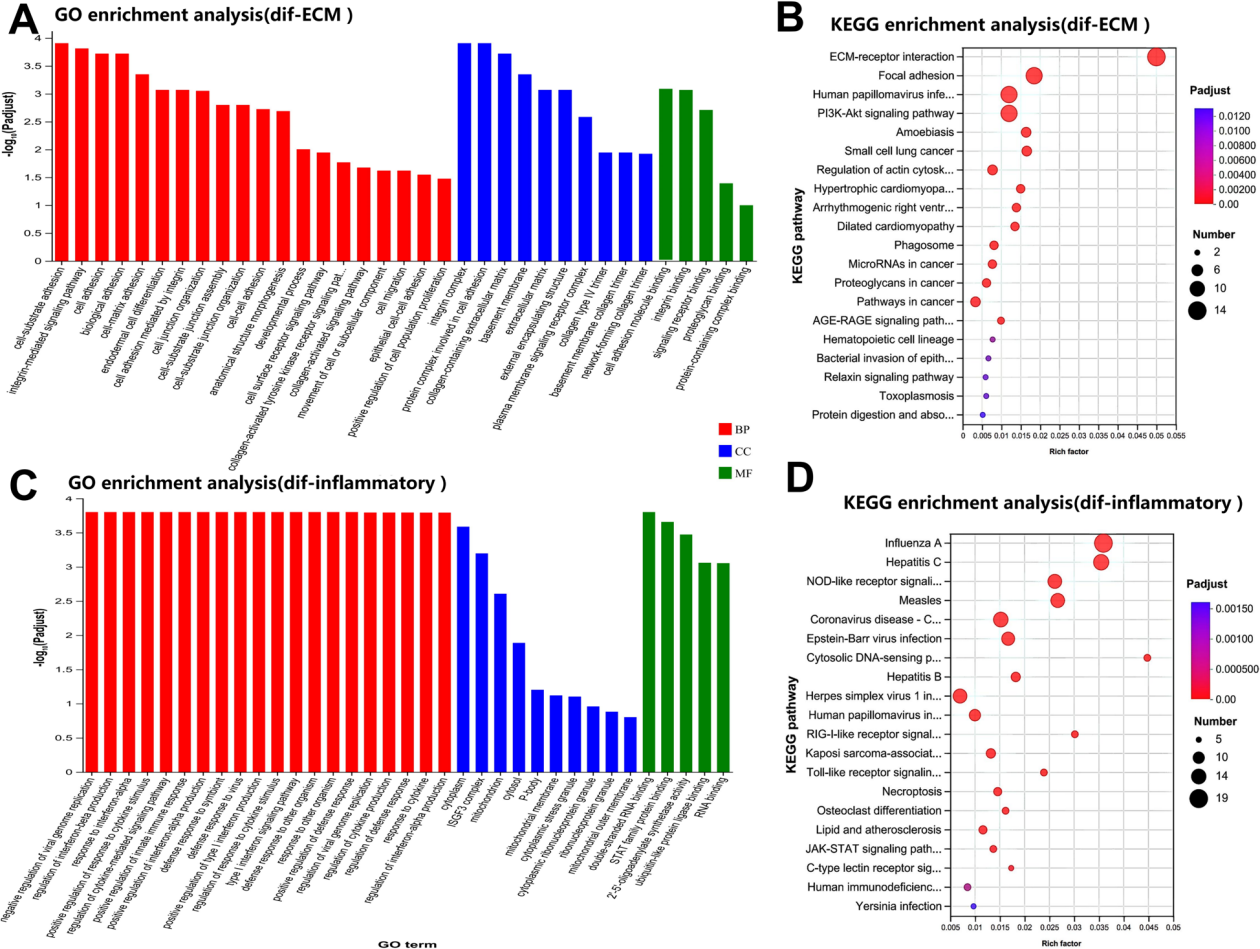
**GO annotation and KEGG enrichment of the downregulated dif-mRNA interactions with the four extracted modules and the upregulated dif-mRNAs related to antiviral effects.** The top 40 GO terms and the top 20 KEGG pathways are shown. **A**, **C** The height of the histogram indicates the significance level of the dif-mRNAs enriched under a specific GO term. **B**, **D** The rich factor in the KEGG scatter plot indicates the proportion of dif-mRNAs enriched in a specific pathway. The size and colour of the solid circles represent the number of enriched dif-mRNAs and the significance of enrichment, respectively.

The dif-mRNA-based PPI network comprised 798 nodes and 2301 interaction pairs (Additional file 8). Four distinct modules in the PPI network were identified: the ECM‒receptor interaction in ECM‒receptor‒interaction module A, the antiviral effect in module B, the inflammatory response in module C, and protein binding in module D (Figure 8). We conducted GO enrichment and KEGG analyses of the dif-mRNAs among the selected modules. The enrichment analysis results revealed that the downregulated dif-mRNAs primarily regulate cellular processes by interacting with specific receptors of ECM molecules, thereby initiating signal transduction pathway cascades that affect key biological events such as differentiation, proliferation, migration, and survival (Figure 9A and Additional file 9). According to the BP, CC, and MF ontologies, the upregulated dif-mRNAs were associated primarily with the viral defence response, the inflammatory response, and protein binding (Figure 9C and Additional file 10). Taking module A as an example, there were 15 nodes and 55 interaction pairs, among which COL4A1, FRAS1, COL4A2, TNC, LAMC2, FN1, LAMA3, ITGA8, ITGB5, ITGA5, LAMA4, ITGA9, FREM1 and thrombospondin-4 (THBS4) presented downregulated expression. The expression levels of numerous genes involved in antiviral and inflammatory responses were significantly increased in OLMECs infected with BTV-1. These include RNASEL, which encodes the IFN-regulated 2-5a system, as well as IFIT2, IFIT3, IFIT5, IFIH1, ISG15, IFN-a-inducible protein 6 (IFI6), bone marrow stromal cell antigen 2A (BST-2A), BST-2B, and IFN regulatory factors 3 and 7 (IRF3 and IRF7), which modulate the effects of IFN-α. For significance, the top 20 KEGG pathways of DIF-mRNAs involved in ECM-receptor interactions and antiviral effects in the module were selected for presentation. Among these pathways, dif-mRNAs for the ECM were enriched mainly in ECM-receptor interaction, focal adhesion, human papillomavirus infection, the PI3K-Akt signalling pathway, amoebic disease, small cell lung cancer, actin cytoskeleton regulation, hypertrophic cardiomyopathy, and arrhythmic right ventricular cardiomyopathy and cancer (Figure 9B and Additional file 11). Differential expression of miRNAs involved in antiviral responses was predominantly observed in influenza A, hepatitis C, the NOD-like receptor signalling pathway, measles, coronavirus COVID-19, Epstein virus infection, hepatitis B, and herpes simplex virus type 1 infection (Figure 9D and Additional file 12).

### Validation of dif-mRNAs by qRT-PCR

**Figure 10:**
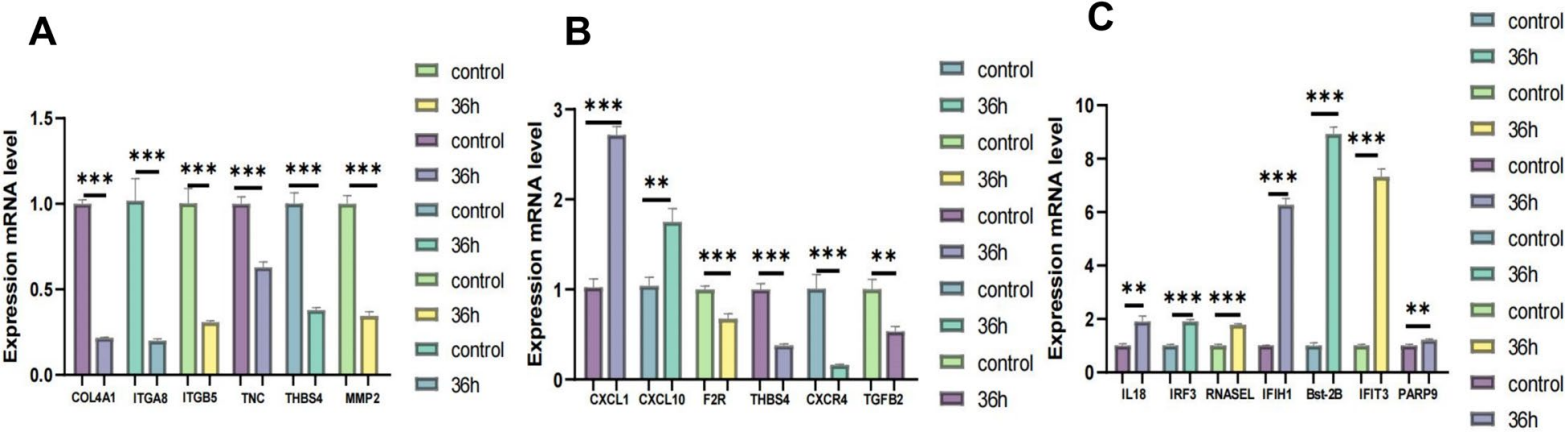
**Verification of the expression levels of A** ECM-dif-mRNAs, **B** inflammatory reaction-dif-mRNAs, and **C** antiviral immunity-dif-mRNAs using qRT-PCR, where qRT-PCR validation results are displayed as mean log_2_ (fold change) ± SD with error bars from three independent experiments. The difference between the indicated groups was significant (*p < 0.05) or very significant (**p < 0.01 and ***p < 0.001).

Verification of dif-mRNA expression was conducted using qRT-PCR. Six mRNAs from the ECM module, six from the inflammatory response module, and seven from the antiviral immune module were selected for qRT-PCR validation in different modules within the PPI network. The infected and control groups showed significant differences. Cluster analysis of dif-mRNA was employed to identify genes associated with the ECM, inflammatory response, and antiviral immunity. Notably, the downregulation of the expression of COL4A1, ITGA8, ITGB5, TNC, THBS4, and MMP2 in the ECM was significantly different (*p* < 0.001) (Figure 8A). The expression levels of the genes associated with antiviral immunity were significantly increased (*p* < 0.01), as shown in Figure 10C. With respect to the inflammatory response, in addition to the upregulated expression levels of CXCL1 and CXCL10, the expression levels of other inflammatory genes were markedly downregulated (*p* < 0.01), as illustrated in Figure 10B.

## Discussion

BTV pathogen-host interactions and their pathogenic mechanisms are highly important for understanding the mechanisms of pathogenesis and prevention. Moreover, the BTV NS4 protein is a type Ι IFN signalling antagonist and determinant of virus virulence [33]. Furthermore, BTV induces a large panel of clinical manifestations ranging from asymptomatic infection to lethal haemorrhagic fever [7]. The gross and histopathological lesions of BT are consistent with a pathogenesis that involves injury to blood vessels, which in turn leads to increased permeability of the affected vessels as well as haemorrhage, thrombosis, and ischemic tissue necrosis (infarction) [34]. Vascular injury in BT has been attributed to direct virus-mediated injury of the EC. However, the RNA expression profiles and biological processes involved in the response of lung ECs to BTV infection have not been comprehensively elucidated. As an increasingly popular method to detect genome-wide gene expression, the combination of expression profile data and bioinformatic analyses has become an effective modality for identifying potential biomarkers and key pathways in various pathogen infections. In this study, to gain an in-depth understanding of BTV infection and vascular injury pathogenic mechanisms, we determined the differential expression profiles of mRNAs, miRNAs, lncRNAs, and circRNAs in OLMECs infected with BTV-1 through high-throughput whole-transcriptome sequencing. Furthermore, we analysed the GO terms and signalling pathways enriched by dif-RNAs, predicted the potential interactions between dif-RNAs, and verified the sequencing results of some dif-RNAs using qRT-PCR. RNA-seq technology is a high-throughput sequencing technique that can be used to determine the expression profiles of differentially expressed mRNAs, miRNAs, lncRNAs, and circRNAs in OLMECs infected with BTV-1. We performed GO and KEGG enrichment analyses on these differentially expressed RNAs and constructed a protein–protein interaction (PPI) network to gain deeper insights into the biological processes and molecular mechanisms associated with BTV infection in host cells, as well as the antiviral defence mechanisms of the host.

Previous studies have shown that autophagy and apoptosis gradually occur at 24 hpi [35–37]. In addition, IFN levels decrease and become undetectable at 24 hpi [38]. Because of the significant expression of the anti-host IFN immune protein, the pathogen‒host interaction and its pathogenic mechanism were studied in depth (Figure 1). Hence, we selected OLMECs infected with BTV-1 at 36 hpi as samples for whole-transcriptome sequencing to clarify the transcription profile changes induced by BTV infection. In our study, we obtained more than 1000 dif-RNA samples, in line with previous findings of BTV-16 infection in PBMCs [39] and BTV-1 infection in OA3. Ts cells [8]. In BTV-16-infected PBMCs, the expression of dif-RNAs involved in the immune response and antiviral defence, such as ISG20 and IFITI, significantly increased. Similar results were observed in BTV-1-infected OA3. Ts cells. In this study, the expression of ENSOART00020013513 (IFIT3), ENSOART00020021906 (ISG15), and ENSOART00020009437 (EPSTI1) was also found to be upregulated (Figure 3A and Additional file 3). Additionally, differential gene transcripts related to IFN were identified, including ENSOARG00020012420 (IGSF8), ENSOARG00020021421 (IFI35), ENSOARG00020003417 (RNASEL), and ENSOARG00020008889 (IFIT5), an inhibitor of IFN-α effects; ENSOARG00020010727 (IFIH1) and ENSOARG00020021024 (IFI6), an IFN-A-induced protein 6; and the bone marrow stromal antigen 2-related genes ENSOARG00020010960 (BST-2A) and ENSOARG00020010913 (BST-2B), along with ENSOARG00020009726 (IRF3) and ENSOARG00020014727 (IRF7) (Additional file 13). The sequencing results from BTV-1 infection in ST cells [35] and BTV-16 infection in PBMCs [39] were similar to those of oar-miR-29b and oar-miR-493-3p (Figure 3B and Additional file 14).

Through functional analysis, this study revealed significant enrichment in canonical signalling pathways, including the Ras, TNF, TGF-β, PI3K-AKT, MAPK, and JAK-STAT pathways (Figure 5). The cytosolic DNA-sensing pathway represents a highly conserved defence mechanism against viral infections [13]. STING generated in the cGAS‒STING pathway recruits TBK1 and IRF3 after phospholipid cleavage [40, 41]. Consequently, following translocation to the cell nucleus, IRF3 performs its transcriptional function by facilitating the expression of immune-stimulating genes (ISGs) and IFN-I [42–44]. In the BTV-infected group, the expression levels of genes such as IRF3, STAT1, BST-2, and others within the type I IFN signalling pathway (GO:0060337) were significantly upregulated, highlighting the crucial role of the type I IFN immune response in host cell resistance against BTV infection (Figures 4A and 7C; Additional file 1). In addition to the aforementioned classical signalling pathways, this study revealed significant enrichment of dif-mRNAs in several signalling pathways associated with viral infections, including Epstein–Barr virus, human T-cell leukemia virus 1, and herpes simplex virus 1 infections. Taking the EB pathway as an example, 55 dif-mRNAs were identified, comprising 51 upregulated and four downregulated dif-mRNAs (Additional file 1). The 51 upregulated dif-mRNAs included CXCL10 (ENSOART00020007022), which plays a significant role in the immune response (GO:0006955), and ENSOARG00020009445 (RIG-1), which is involved in the RIG-I-like receptor signalling pathway. By downregulating four dif-mRNAs, ENS0ARG00020025633 (IKBKB) was identified as a pivotal regulator of the innate immune response (Additional file 1).

Many bioactive molecules within the ECM can directly or indirectly influence cellular structure and function [45]. The GO classification of DETs revealed enrichment of immune system processes, inflammatory processes, binding processes, ECM components, and cellular processes (Figure 5). The TGF-β pathway regulates the transcription of inflammatory factors and ECM genes [46]. In OLMECs infected with BTV, the expression levels of genes located downstream of the TGF-β pathway, such as TGFB1 (ENSOARG00020010032), COL4A1 (ENSOART00020005366), FN1 (ENSOART00020039176), and THBS4 (ENSOARG00020004359), were significantly downregulated (Figure 8A). These findings suggest that BTV can suppress the transcription of ECM genes by regulating the TGF-β pathway. Notably, this study revealed significant enrichment of dif-miNRA target genes in pathways associated with the ECM, including the ECM (GO:0031012), collagen-containing ECM (GO:0062023), and ECM‒receptor interaction (map04512) (Figures 4 and 5), via GO and KEGG analyses. However, upon BTV-1 infection, OA3. In Ts cells, there was a predominant enrichment of dif-miRNA target genes in GO terms related to the cytoplasm, according to CC ontology, indicating a substantial difference [8]. The downregulated dif-mRNAs were enriched mainly in ECM-related pathways under the BP, CC, and MF ontologies in the GO analysis (Figure 8B). In this study, the GO and KEGG analyses revealed significant enrichment of ECM (GO:0031012), collagen-containing ECM (GO:0062023), and ECM-receptor interaction (map04512) in OLMECs infected with BTV, as illustrated in Figures 4 and 5. On the basis of the aforementioned analysis, it can be inferred that following BTV infection, OLMECs exhibit significantly elevated expression of genes associated with the ECM. BTV infection may induce alterations in the ECM of host cells, thereby influencing intracellular signal transduction through interactions between bioactive substances present within the ECM and their corresponding receptors. Consequently, the OLMECs become more susceptible to BTV.

Infection with pulmonary microvascular ECs constitutes the fundamental mechanism underlying disease manifestation in sheep infected with BTV [13]. Because of the dynamic equilibrium between vascular wall cells and the ECM, the invasion of pathogens disrupts this delicate balance, thereby compromising the ability of microvessels to combat diseases, resulting in oedema, inflammation, bleeding, and infection [47]. In the ECM, large molecules such as COL4A1, TNC, and THBS4 exhibit binding affinity toward integrin family receptors, thereby actively participating in the intricate regulation of cellular signalling [45]. Among them, THBS4, a glycoprotein with adhesive properties, plays a crucial role in facilitating cellular interactions with the ECM. It is intricately involved in various cellular processes, including proliferation, migration, adhesion, attachment, and regulation of vascular inflammation. THBS4 can bind to structural ECM proteins and effectively modulate ECM remodelling in response to tissue damage, thereby contributing to adaptive tissue repair [47]. The results of this study revealed downregulation of the expression of ENSOART00020005366 (COL4A1), ENSOART00020039176 (FN1), ENSOART00020014404 (ACAN), MSTRG.11050.3 (DCN), ENSOART00020025703 (TNC), and ENSOARG00020004359 (THBS4) (Additional file 1). Additionally, real-time fluorescence quantitative PCR revealed significant decreases in the expression of COL4A1, TNC, and THBS4 (Figure 10A). These findings suggest that BTV infection can affect ECM remodelling processes, thereby impeding microvascular EC healing. The primary role of the ECM is facilitated by integrins, which facilitate the transmission of signals between the extracellular environment and intracellular pathways [48, 49]. In this study, the GO analysis revealed a significant enrichment of integrin binding (GO:0005178), cell adhesion mediated by integrin (GO:0033627), and positive regulation of cell adhesion mediated by integrin (GO:0033630) (Figure 4). These findings highlight the crucial role of integrin signalling in oligodendrocyte precursor cells following BTV infection. As one of the primary components initially exposed to viral infection, the ECM releases a danger signal upon degradation by matrix metalloproteinases (MMPs), thereby triggering an immune response in the host [50]. MMPs play indispensable roles in facilitating neutrophil infiltration into inflammatory sites and controlling viral replication. In this study, the expression of MMP1, MMP16, and MMP2 was downregulated in the BTV-infected group (Additional file 1). Notably, we did not validate these differential genes at the protein level. BTV invasion may suppress the expression of MMP genes as a mechanism to inhibit ECM degradation and prevent the release of danger signals, thereby ensuring its successful replication and proliferation.

The CXC family is considered a pivotal factor in the infiltration of leukocytes and the activation of the host inflammatory response. Furthermore, they can interact with the ECM following translational modifications to regulate inflammation and immune reactions. For example, CXCL14, a constituent of its family, can facilitate the transmigration of inflammatory cells into ECs by modulating the thickness of the ECM glycogen layer [51]. In this study, the significant downregulation of COL4A1 and COL4A2, located within the ECM (GO:0031012), may have contributed to the notable enrichment of the inflammatory response (GO:0006954) in our GO analysis (Figure 5 and Additional file 1). Furthermore, we observed high expression of ENSOART00020007022 (CXCL10) and ENSOART00020004532 (CXCL1), which are located within the inflammatory response (GO:0006954) and extracellular region (GO:0005576) categories, suggesting their crucial role in positively regulating the inflammatory response of the host. This finding may explain the significant enrichment of leukocyte migration through ECs in the ceRNA network, as indicated by KEGG analysis (Figure 7B). The chemokine receptor CXCR4 serves as a specific receptor for CXCL12, exerting a potent chemotactic effect on lymphocytes. Furthermore, animal models have demonstrated that homologous agonists of CXCR4 can confer pulmonary protection by mitigating lung damage and promoting the generation of endothelial progenitor cells [52]. The findings of this study suggest that following BTV infection, OLMECs may impede the repair process of EC damage by significantly downregulating the expression of CXCR4 (Additional file 1). Research findings indicate that BTV-specific T cells isolated from lymphocytes produce IFN-γ upon recognizing presented antigens and participate in the regulation of IL-10, which is involved in inflammation and cellular immunity [53]. Moreover, BTV can induce NLRP3 inflammasome activation, thereby triggering caspase-1 activation and facilitating the generation and release of proinflammatory cytokines, such as IL-18, into the extracellular milieu, consequently leading to inflammation [54]. In this study, transcriptome analysis and fluorescence quantification also revealed significant upregulation of IL-18 expression in the BTV-infected group (Figure 9C). This finding suggests that BTV induces an inflammatory response in host cells by activating the NLRP3 inflammasome, leading to the production and release of IL-18 in OLMECs [55]. A negative correlation has been demonstrated between TGF-β and CXCL1 expression [56]. In accordance with our findings, infection of OLMECs with BTV resulted in the upregulation of ENSOART00020004532 (CXCL1) expression, whereas the expression of TGFB1 was significantly downregulated (Additional file 1.). These findings suggest that BTV infection in OLMECs can induce the activation of the CXC family and NLRP3-related genes while simultaneously inhibiting the expression of TGF-β and ECM-related genes, ultimately leading to pneumonia.

This study conducted a systematic analysis of the transcriptome of BTV-infected OLMECs, outlining why sheep OLMECs are susceptible to BTV infection. Moreover, this study focused on revealing the biological processes involved in BTV infection-induced lung inflammation, offering new research perspectives on the interaction between the BTV and its host. Notably, following BTV invasion of OLMECs, the ECM acts as a central hub for signal regulation, including growth factor receptors and integrins, controlling the interaction between BTV and host cells through ECM degradation and remodelling. Unfortunately, our findings did not demonstrate the involvement of hyaluronic acid, a pivotal regulator of inflammation in the ECM. Moreover, restricting our analysis to only the 36-h time point may be inadequate for comprehensively understanding the dynamic alterations in the ECM after BTV infection and their effects on invasion and the host response. Significant variations in transcriptome changes were observed among different cell types during the investigation of BTV infection, potentially influencing our analysis. We subsequently leveraged the fact that the ECM is located upstream of the PI3K-AKT pathway and that its interaction with integrins activates PI3K-ATK, which ultimately regulates apoptosis, migration, and immune cell activation. This mechanism should be further investigated.

## Supplementary Information

**Additional file 1. List of all differentially expressed RNAs in the BTV-infected group.**

**Additional file 2. Hierarchical clustering analysis results for the differential RNA between OLMECs infected with BTV-1 and the control group.**

**Additional file 3. GO annotation analysis of differentially expressed RNA in the BTV-infected group.**

**Additional file 4. GO annotation analysis of dif-miRNA target mRNAs in the BTV-infected group.**

**Additional file 5. GO annotation analysis of dif-lncNRA target mRNAs in the BTV-infected group.**

**Additional file 6. GO annotation analysis of the host mRNAs of circRNAs.**

**Additional file 7. Analysis of ceRNA relationships.**

**Additional file 8. The dif-mRNA-based protein‒protein interaction (PPI) network was composed of 798 nodes and 2301 interaction pairs.**

**Additional file 9. GO annotation analysis of differentially expressed transcripts related to ECM.**

**Additional file 10. GO annotation analysis of differentially expressed transcripts involved in antiviral responses between the host and pathogen.**

**Additional file 11. KEGG enrichment analysis of differentially expressed transcripts related to ECM.**

**Additional file 12. KEGG enrichment analysis of differentially expressed transcripts associated with antiviral effects in the host.**

**Additional file 13. Interferon-associated dif-RNAs.**

**Additional file 14. Hierarchical clustering analysis results for differentially expressed miRNAs between the BTV-1-infected OLMECs and control OLMECs.**

## Data Availability

The datasets used and/or analysed during the current study are available from the corresponding author upon reasonable request.
