# Supplementary figures and images for: Whole-transcriptome analyses of ovine lung microvascular endothelial cells infected with bluetongue virus
Source: Vet Res. 2024 Sep 27;55:122. doi: 10.1186/s13567-024-01372-0 (PMC11438077; doi:10.1186/s13567-024-01372-0)

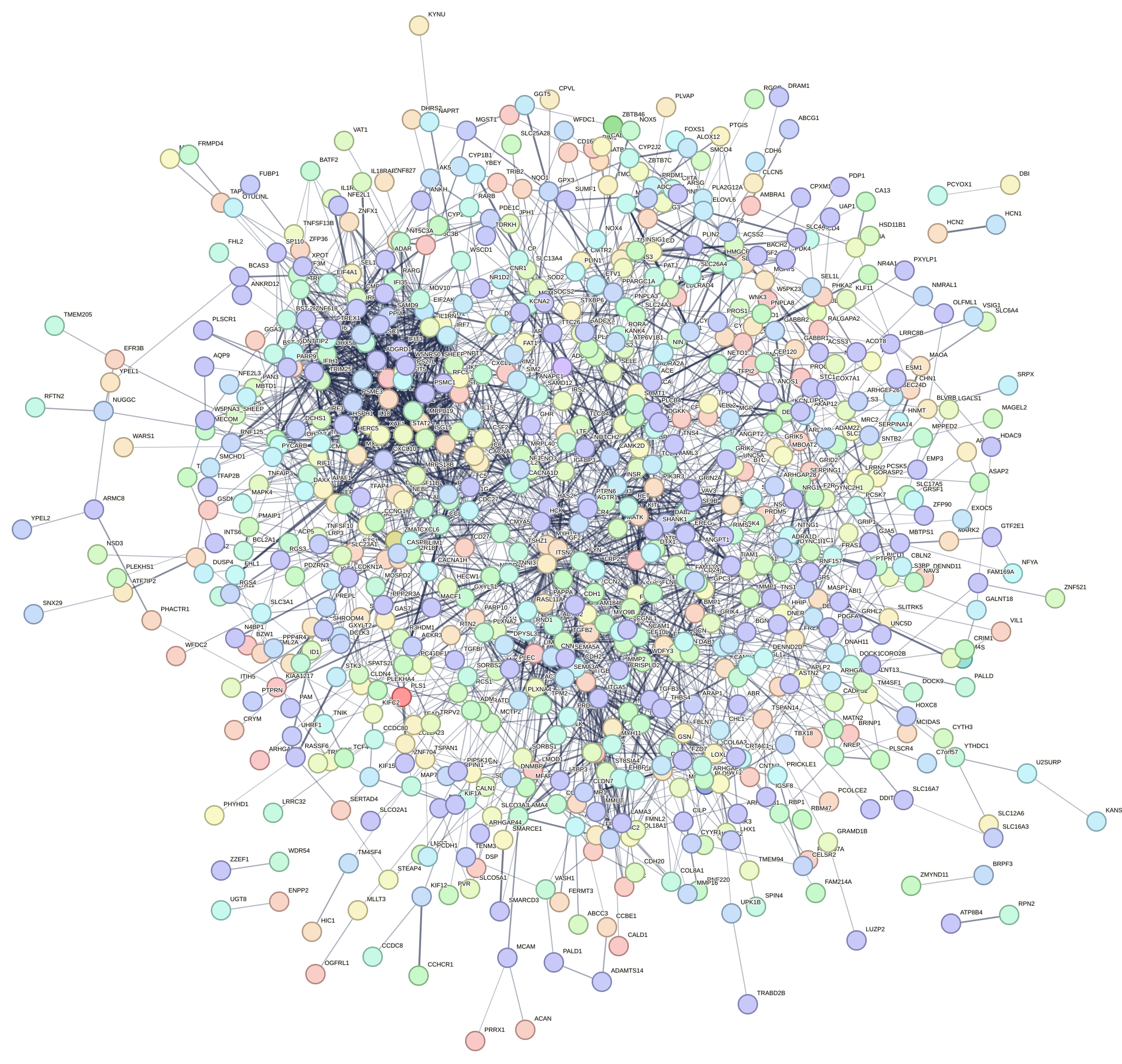

Supplement: Supplementary file 8 — Additional file 8. The dif-mRNA-based protein‒protein interaction (PPI) network was composed of 798 nodes and 2301 interaction pairs. [file 13567_2024_1372_MOESM8_ESM.png]
